# Quality inspection and result analysis of the spirometer calibration cylinder

**DOI:** 10.1186/s12890-022-02010-1

**Published:** 2022-06-04

**Authors:** Zhongping Wu, Yongyi Peng, Kuiqing Lin, Ruibo Huang, Jinping Zheng, Yi Gao

**Affiliations:** National Center for Respiratory Medicine, State Key Laboratory of Respiratory Disease, National Clinical Research Center for Respiratory Disease, Guangzhou Institute of Respiratory Health, First Affiliated Hospital of Guangzhou Medical University, Yanjiang Road 151, Guangzhou, 510120 People’s Republic of China

**Keywords:** Volume calibration syringes, Quality control, Volume accuracy, Leak test

## Abstract

**Background:**

To understand the accuracy of volume calibration syringes used in China and compare the difference between new and old volume calibration syringes, technical testing was performed on volume calibration syringes in clinical lung function instruments.

**Materials and methods:**

A standard validator device (Model 1180, Hans Rudolph, USA) was used to perform leak testing and volume accuracy testing for calibration syringes. Sixteen volume calibration syringes from 8 brands (CareFusion in Germany, Vyaire in Germany, Yaeger in Germany, Vitalograph in the United Kingdom, MGC Diagnostics in the United States, U-Breath in Zhejiang, China, Wendi in Ningbo, Zhejiang, and Boya in Ningbo, China) were tested.

**Results:**

A total of 75% (12/16) of the volume calibration syringes passed the pressure decay leak test, 69% (11/16) of the volume calibration syringes passed the volume accuracy and repeatability test, and 56% (9/16) passed both tests; there was no significant difference in the total passing of the new and old volume calibration syringe quality tests (*P* > 0.05).

**Conclusions:**

A standard validator device should be used for both leakage tests and volume accuracy and repeatability tests to ensure the reliability of volume calibration syringes. It is suggested that the quality verification of volume calibration syringes should be regularly conducted to ensure the accuracy of the pulmonary function tests.

## Introduction

A pulmonary function test is a medical measurement technology, and the quality of the test equipment is one of the key links that affect the quality control of a test. In the single-breath carbon monoxide lung diffusion standard released by the American Thoracic Society (ATS)/European Respiratory Society (ERS) in 2017 [1] and the updated version of the spirometer examination guidelines released in 2019 [2], it is proposed that all spirometers must satisfy the specified technical standards and quality control standards [3]. During the quality control process, the spirometer should be calibrated by the volume calibration syringe each time, and the error should be within ± 2.5% (the instrument error of lung function should be within ± 2%; the volume calibration syringe error should be within ± 0.5%). After calibration, an accurate calibration coefficient can be obtained. The result of a spirometer check is obtained by multiplying the value measured by the sensor by a calibration factor [4]. The volume calibration syringe is an important part of the quality control process of the spirometer and the accuracy of the spirometer data. The accuracy of the volume calibration syringe directly affects the accuracy of spirometer data and consequently the interpretation of clinical pulmonary function results. In clinical practice, standard respiratory simulators and volume calibration syringes can be used for spirometer calibration, while manual push–pull volume calibration syringes are the basic method [5, 6].

The ATS/ERS single-breath carbon monoxide lung diffusion standard (2017) and updated version of the spirometry guidelines (2019) propose that the volumetric accuracy of the volume calibration syringes should be tested monthly but do not suggest which equipment should be used for calibration or detailed calibration procedures. In 2011, Madsen [7] conducted volume tests on 11 volume calibration syringes of 6 brands and different ranges using a weighing meter, water level gauge and thermometer. The calibration syringe discharged water from the water level gauge with the maximum stroke volume and subsequently determined the volume of water according to the water temperature and weight to represent the volume of the calibration syringe. The steps of this method are very tedious and require repeated switching and recording among different measuring instruments. Additionally, a test process using discharged water must be repeated many times. The calculation results are more troublesome and prone to error. The leakage test is estimated by recording the volume and the corresponding time several times, i.e., by applying a pressure of 4 hPa to the rod of the calibration cylinder, weighing the weight of the water discharged from the water level gauge with a weighing meter, and weighing the discharged water after keeping it for 30 min. The amount of air leakage of the volume calibration syringe is determined by the change in weight, which is subject to human interference and easy operation errors, which may cause inaccurate data.

Because the calibration method was unclear or too complex, some medical institutions never performed calibration verification for volume calibration syringes. We have learned that Hans Rudolph has a validator device that can perform volume accuracy tests and leak tests on volume calibration syringes. Therefore, in this study, a standard validator device was used to conduct leak tests and volume accuracy tests on the volume calibration syringes of spirometers commonly used in clinical practice. These measurements were conducted to understand the performance of laboratory volume calibration syringe equipment, standardize calibration and detection methods of volume calibration syringes and compare the new and old volume calibration syringes in the clinical work of the pulmonary function.

## Objects and methods

### Object

The research objects were 16 new and old volume calibration syringes of 8 different brands from the Pulmonary Function Testing Center of the State Key Laboratory of Respiratory Diseases, including 8 brand-new and unused volume calibration cylinders (CareFusion, Germany; Vyaire, Germany; Yaeger, Germany; Vitalograph, UK; MGC diagnostics, USA; U-Breath, Zhejiang, China; Wendi, Zhejiang, China; and Boya, Zhejiang, China) and 8 old volume calibration cylinders with a service life of 1–5 years (1 set from CareFusion Corporation of Germany, 5 sets from Vyaire Corporation of Germany, 1 set from Yaeger Corporation of Germany, and 1 set from U-Breath Corporation of China). The new volume calibration syringes were named NEW-D1–D8, wherein the volume of NEW-D1 was 1000 mL, the volume of NEW-D2–D7 was 3000 mL, and the volume of NEW-D8 was 100 mL. The old calibration tubes are named OLD-D1–D8, wherein the volume of OLD-D1–D7 is 3000 mL; OLD-D8 is 100 mL.

### Experimental equipment

**Fig. 1:**
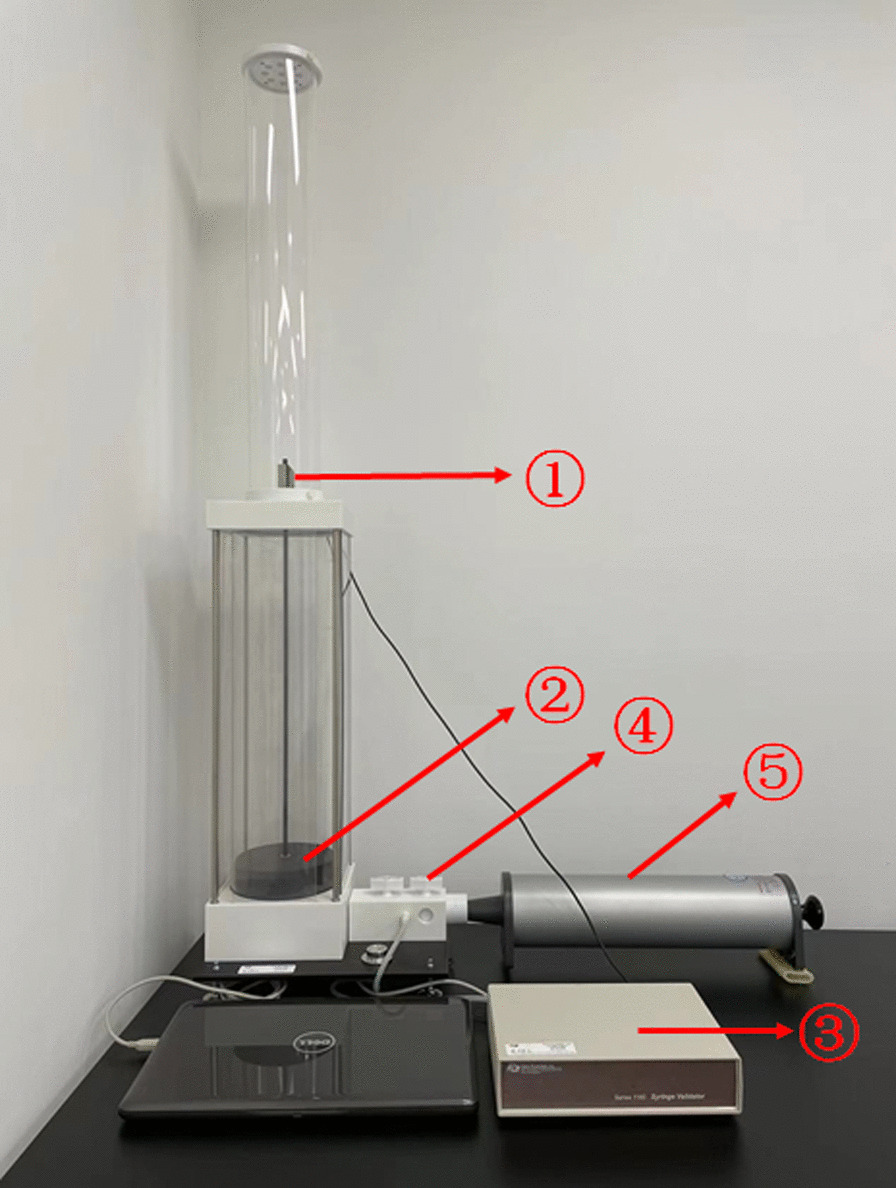
Operation diagram of the volume calibration syringe of the 1180 capacity calibration cylinder verifier from Hans Rudolph Company in the United States. *Note*: ① optical encoder, ② graphite piston, ③ controller, ④ rotary valve, and ⑤ volume calibration syringe to be verified

The standard validator device (Hans Rudolph, USA) is a precision device that requires careful setup and proper use to assure accuracy of the performed measurements. The system consists of three main components: the main syringe validator volume measuring device, the instrumentation and control box and a computer system (Fig. 1). After the volume calibration syringe was connected, the benchmark rod of the volume calibration syringe was pushed and pulled, so the gas in the volume calibration syringe entered and left the volume measuring device to make the graphite piston in the glass tube rise and fall to detect the volume accuracy. The leak test used a controller to pressurize the volume calibration syringe to a low pressure (20 cmH2O). Then, after a period of time (approximately 30 s), the change in air pressure was measured to determine how much leakage occurred during the test.

### Methods and steps

*Leak test* Before testing, each volume calibration syringe and standard validator device were placed in the same environment for at least 1 h; the validator software was started, and the devices were preheated for 30 min. Then, the volume calibration syringe rod was completely elongated and connected to the coupler of the validator, and attention was given to the tightness. The test information was entered into the software, and the test parameters and environmental conditions were selected. The “leak test” button was clicked to start the volume test. The software was employed to rotate the plug valve to a specified position and extend the rod to the end. The controller was used to pressurize the volume calibration syringe to the test pressure (default 20 cmH_2_O). The pressurization continued to maintain the pressure of the entire system constant for 30 s, and the controller was used to check whether the pressure was leaking. A report was saved and generated after the test.

*Volume accuracy test* Before testing, each volume calibration syringe and standard validator device were placed in the same environment for at least 1 h. The verifier software was started, and the devices were heated for 30 min. The volume calibration syringe rod was fully extended and connected to the verifier; attention was given to the tightness. The test information was entered into the software, and the test parameters and environmental conditions were selected. The “start verification” button was clicked to start the volume test. The software prompts the user to rotate the plug valve to a specified position. When the progress indicator light on the software was green and ready, the rod of the volume calibration syringe was manually pushed, and the graphite piston in the glass tube was raised, which was the propulsion stroke. When the indicator light turned yellow, the signal could be restored. Then, the rod could continue to be artificially elongated, while the graphite piston in the glass tube dropped, which was a pullback stroke. When the green signal appeared, the push and pull stroke was repeated at least 3 times, as indicated by the indicator, until the measurement averages and range values were obtained. When the test was complete, the stop validation button was clicked, and a test report was saved and generated.

### Evaluation criteria

*Leakage test* A test in which the controller is pressurized to the test pressure, and a pressure decay measurement is performed to check whether there is a change in volume. The target leakage should be 0 mL/min, and the actual allowable leakage error should be within ± 1 mL/min.

*Volume accuracy test* The difference between the target value and the measurement average was used to calculate the error, which should be within ± 0.5% of the target volume. The range of multiple measurements (maximum–minimum) was used to calculate the repeatability, which should be within ± 0.05% of the target volume. The volume was 3000.0 mL, the average error was within ± 15 mL, and the volume error repeatability was within ± 1.5 mL. The volume was 1000.0 ml, the average error was within ± 5 mL, and the volume error repeatability was within ± 0.5 mL. The target volume was 100.0 mL, the average error should be within ± 0.5 mL, and the volume error repeatability should be within ± 0.05 mL.

### Statistical methods

SPSS 21.0 software was used for statistical analysis. The quantitative data with a normal distribution are described as the mean ± standard deviation, and the passing rates of the new and old volume calibration syringes were compared the χ^2^ test. *P* < 0.05 was considered statistically significant.

## Results

A total of 75% (12/16) of all the volume calibration syringes passed the pressure decay leak test, 69% (11/16) of the volume calibration syringes passed the volume accuracy and repeatability test, and 56% (9/16) passed both tests; there was no significant difference in the total passing of the new and old volume calibration syringe quality tests (χ^2^ = 0.3, *P* > 0.05). The pass rates of the new volume calibration syringe leak test and volume accuracy test single test were 88% and 75%, respectively. The pass rate of the leak test and volume accuracy test using a certain-year-limited standard cylinder were both 63% (Table 1).

**Table 1:** Results of the leak test and volume accuracy test for 16 volume calibration syringes

The evaluation index	NEW-D1	NEW-D2	NEW-D3	NEW-D4	NEW-D5	NEW-D6	NEW-D7	NEW-D8	OLD-D1	OLD-D2	OLD-D3	OLD-D4	OLD-D5	OLD-D6	OLD-D7	OLD-D8	Overall pass rate/%
Use fixed number of years	0	0	0	0	0	0	0	0	2.5	2.5	1	1.5	3	3	2	5	
*Leak test*																	
The target volume (mL)	1000.0	3000.0	3000.0	3000.0	3000.0	3000.0	3000.0	100.0	3000.0	3000.0	3000.0	3000.0	3000.0	3000.0	3000.0	100.0	
Objective to reveal (mL/min)	0.0	0.0	0.0	0.0	0.0	0.0	0.0	0.0	0.0	0.0	0.0	0.0	0.0	0.0	0.0	0.0	
Actual average leakage (mL/min)	0.1	0.2	0.9	0.5	2.5	0.0	0.8	0.3	0.3	0.1	0.4	0.2	1.0	1.9	—	0.1	75
Reveal the mean error value (mL/min)	0.1	0.2	0.9	0.5	2.5	0.0	0.8	0.3	0.3	0.1	0.4	0.2	1.0	1.9	—	0.1	75
Whether through (Y/N)	Y	Y	Y	Y	N	Y	Y	Y	Y	Y	Y	Y	N	N	N	Y	75
*Volume accuracy test*																	
The target volume (mL)	1000.0	3000.0	3000.0	3000.0	3000.0	3000.0	3000.0	100.0	3000.0	3000.0	3000.0	3000.0	3000.0	3000.0	3000.0	100.0	
Average volume measurement (mL)	992.2	2994.7	2996.9	3003.0	2990.9	2995.7	3001.2	96.9	2994.4	2998.6	2992.5	2990.6	2995.4	2998.9	2979.2	92.1	88
Volume mean error range (mL)	0.5	0.4	0.6	0.5	0.8	1.4	0.4	0.4	1.5	0.6	1.0	1.2	0.4	1.8	13.5	0.9	69
Volume mean error (mL)	− 7.8	− 5.3	− 3.1	3.0	− 9.1	− 4.3	1.2	− 3.1	− 5.6	1.4	− 7.5	− 9.4	− 4.6	− 1.1	− 20.8	− 7.9	88
Whether through (Y/N)	N	Y	Y	Y	Y	Y	Y	N	Y	Y	Y	Y	Y	N	N	N	69
Two tests whether through (Y/N)	N	Y	Y	Y	N	Y	Y	N	Y	Y	Y	Y	N	N	N	N	56

“—” indicates that the leak test could not be completed due to serious gas leakage during the leak test

## Discussion

Pulmonary function testing is a key technology for diagnosing and treating respiratory diseases, among which spirometry is an important health technology for the early screening and intervention management of chronic respiratory diseases in medical institutions [8]. A pulmonary function tester is the main equipment for pulmonary function testing. The quality control and accuracy of measurement values of this device directly affect the interpretation of results and clinical disease diagnosis. As an important accessory attached to the spirometer, the accuracy of the tester directly affects the measurement of lung function and validity of the value. According to China's pulmonary function testing guidelines and ATS/ERS pulmonary function testing standards, each instrument must use a volume calibration syringe for volume calibration every day with weekly linearity verification. Thus, each spirometer must be simultaneously tested and should be equipped with volume calibration syringes. Therefore, calibration cylinders of volume calibration syringes, especially many Chinese independent brands, have also flooded into the international medical market. However, there is still a lack of research on whether the performance of these calibration cylinder devices can satisfy clinical needs. This study found that the spirometry standard updated by ERS in 1993 suggested that a calibrated calibration cylinder should be used, and the volume calibration syringes should be accurate to within 25 mL [9]. However, the 1994 ATS guideline did not provide calibration specifications for volume calibration syringes [10]. The 2005 ATS/ERS standard states “The accuracy of volume calibration syringes must be within ± 15 mL or ± 0.5%”, but no specific reason is provided [11]. The volume should be 3 L, but the rationale for that is unclear. In this study, the leak test and volume accuracy test were used to test the quality of the volume calibration syringes, and the quality of the calibration cylinder was scientifically and objectively evaluated by evaluating the passing conditions of the two tests. This method has high feasibility and is more conducive to clinical application.

In this study, the newly developed capacity calibration cylinder verifier developed by Hans Rudolf of the United States was used to pressurize the air in the volume calibration syringes to determine the amount of air lost during a set period of time, thereby performing a pressure decay leak test. The calibration test of the volume calibration syringes was performed by emptying the air of the calibration cylinder into the “calibration cylinder verifier” [12]. Then, the amount of air introduced and whether it satisfies the specifications of the rated volume calibration syringes are determined. The test is directly generated with the computer software system. As a result, the entire operation is simple and easy to understand, greatly reduces human interference factors and effectively improves the detection accuracy and efficiency.

During the test, several operating points are summarized as follows: (1) The test method of this study directly measures the change in gas volume of the calibration cylinder. Since it is a gas volume, it is necessary to avoid all factors during the standardized operation, such as the impact of the changes in environmental parameters. The volume calibration syringes and standard validator device must be placed under identical environmental conditions for at least 1 h. When the ambient temperature changes more than 2 °C, the test must be repeated; the airflow of the heating and cooling system or any place that generates heat should be kept as far away as possible during the test. During the process, hands should not be placed on the calibration cylinder, as the heat from hands will heat the air and change the volume measurement. (2) The stroke of the same calibration cylinder must not exceed 20 times. Continuously moving the volume calibration syringe will slightly heat it and change the volume reading. After cooling and resting for at least 20 min, testing was continued. (3) The stroke speed of the rod of the push-pull volume calibration syringe should be maintained between 1 and 3 s. The extreme stroke speed (slow or fast) will affect the accuracy. (4) When conducting a leak test, whether the glass tube of the verifier is intact and whether the volume calibration syringe has broken parts or other defects, which may cause cracking or disassembly during the leak test, should be checked. Leak testing will pressurize the calibration cylinder under test, which may cause the defective volume calibration syringes to fly out of the end or to be ruptured.

Using the test method in this study, the volume accuracy and stability of the volume calibration syringes can easily be obtained. The test results indicate that 44% (7/16) of the volume calibration syringe failed the volume accuracy test and/or leak test. The OLD-D7 volume calibration syringe has a long service life and has never been maintained, which results in serious gas leakage. These volume calibration syringes cannot be tested for leaks. There was no difference in the passing rate of the new and old volume calibration syringes (*P* > 0.05); 38% (3/8) of the new volume calibration syringes failed to pass the two tests, which indicates that some brands of equipment manufacturers did not produce sufficiently qualified products, and 50% (4/8) of old volume calibration syringes failed both tests but were still used for clinical testing of spirometers and were never quality tested. Madsen's [7] test results showed that two 1-L volume calibration syringes failed to pass the gas volume test. The 1-L and 0.1-L small-volume calibration syringes (NEW-D1, NEW-D8 and OLD-D8) in our study passed the leak test regardless of whether they were new or old, but they failed to pass the volume accuracy test. The volume calibration syringe is an important tool for the daily calibration of spirometers in clinical medical institutions. If the accuracy and stability of the volume calibration syringes are not up to standard, then inaccurate measurement results of the spirometer will inevitably be obtained. Thus, it is necessary to use a feasible, effective, simple and homogeneous method to calibrate the volume calibration syringes of spirometers, which is an important guarantee to obtain accurate results of clinical pulmonary function tests.

Regarding the capacity volume calibration syringe of the spirometer, the 2019 ATS/ERS pulmonary function testing standard requires a calibration cycle to be performed once a year, and adjustments should be made when necessary. In general, the optimal calibration or verification interval depends on the number of errors in the spirometer data due to volume calibration syringe failure and the cost of the verification test. In a pulmonary function laboratory with a large volume of pulmonary function testing and pulmonary function instruments, the volume calibration syringe will be more frequently used, and the time period for which the volume calibration syringe is worn may also be shortened. With the development of technology, some scholars have conceived intelligent calibration tools for the spirometer volume and flow rate since it only requires five syringe strokes to produce a calibration curve with acceptable volume and flow errors [13]. However, manual push-pull calibration cylinders for spirometer calibration continue to be mainstream. Therefore, whether the verification interval of 1 year is reasonable should be further explored.

The volume calibration syringes verified in this study include new equipment that has been used for many years or has not been used before, which is representative. However, in actual clinical practice, the volume calibration syringes of many pulmonary function examination rooms have been used for many years and may be worn or loosened. The limitations of this study are the limited sample size, inability to evaluate the batch-to-batch variation in volume calibration syringes of the same brand, and lack of tests on other pulmonary function laboratory volume calibration syringes to obtain their real data. These limitations will be supplemented and improved in the next study.

## Conclusions

In summary, the standard validator device should be used to test the leakage, volume accuracy and repeatability to ensure the reliability of the volume calibration syringes. The pulmonary function room of the medical institution should verify the volume calibration syringes at least once a year to ensure the accuracy of the pulmonary function tests.

## Data Availability

All data generated or analyzed during this study are included in this published article.

## Abbreviations

ERS: European Respiratory Society
ATS: American Thoracic Society

## References

[1] Graham BL, Brusasco V, Burgos F (2017). 2017 ERS/ATS standards for single-breath carbon monoxide uptake in the lung. Eur Respir J.

[2] Graham BL, Steenbruggen I, Miller MR (2019). Standardization of spirometry 2019 update. An Official American Thoracic Society and European Respiratory Society Technical Statement. Am J Respir Crit Care Med..

[3] Pulmonary Function Professional Group, Respiratory Diseases Branch of Chinese Medical Association. Guidelines for pulmonary function examination (part 2)-spirometer examination. Chin J Tuberc. Respir. 2014;37(7):481–486.

[4] McCormack MC, Shade D (2007). Spirometer calibration checks: is 3.5% good enough?. Chest.

[5] ISO 26782 (2009). Anaesthetic and respiratory equipment—Spirometers intended for the measurement of time forced expired volumes in humans.

[6] Wu Z, Huang R (2022). Technical performance analysis of different types of spirometers. BMC Pulm Med.

[7] Madsen F (2012). Validation of spirometer calibration syringes. Scand J Clin Lab Invest.

[8] Dongying Z, Yi G (2020). The feasibility and suggestions for the promotion of lung function examination technology in primary medical and health institutions. Chin General Pract.

[9] Quanjer PH, Tammeling GJ (1993). Lung volumes and forced ventilatory flows. Eur Respir J.

[10] Standardization of Spirometry, 1994 Update. American Thoracic Society. Am J Respir Crit Care Med. 1995;152(3):1107–36. 10.1164/ajrccm.152.3.7663792 7663792

[11] Miller MR, Hankinson J, Brusasco V (2005). Standardisation of spirometry. Eur Respir J.

[12] Gilbey JD, Wilson M (2021). Measurement of gas flow and volume. Intensive Care Med.

[13] Cross TJ, Kelley EF, Hardy TA, Isautier JMJ, Johnson BD (2019). The syringe potentiometer: a low-cost device for pneumotachograph calibration. J Appl Physiol.

